# The effect of maintaining high hemoglobin levels on long-term kidney function in kidney transplant recipients: a randomized controlled trial

**DOI:** 10.1093/ndt/gfy365

**Published:** 2018-12-17

**Authors:** Makoto Tsujita, Tomoki Kosugi, Norihiko Goto, Kenta Futamura, Morikuni Nishihira, Manabu Okada, Takahisa Hiramitsu, Shunji Narumi, Kazuharu Uchida, Asami Takeda, Kunio Morozumi, Shoichi Maruyama, Yoshihiko Watarai

**Affiliations:** 1 Department of Transplant Nephrology and Surgery, Kidney Disease Center, Nagoya Daini Red Cross Hospital, Nagoya, Japan; 2 Department of Nephrology, Nagoya University Graduate School of Medicine, Nagoya, Japan; 3 Department of Renal Transplantation, Masuko Memorial Hospital, Nagoya, Japan; 4 Department of Nephrology, Kidney Disease Center, Nagoya Daini Red Cross Hospital, Nagoya, Japan

**Keywords:** correction of anemia, estimated glomerular filtration rate, kidney transplant recipients, randomized controlled trial

## Abstract

**Background:**

Posttransplant anemia may be a major determinant of chronic allograft nephropathy. However, the impact of correcting anemia on graft function remains controversial.

**Methods:**

A 3-year follow-up of an open-label, multicenter, randomized controlled trial involving kidney transplantation recipients examined whether sustained maintenance of target hemoglobin (Hb) concentrations at a high level (12.5–13.5 g/dL, *n* = 64) with either darbepoetin alfa or epoetin beta pegol would slow the graft function decline rate as the primary efficacy endpoint, compared with maintenance of a low Hb concentration (10.5–11.5 g/dL, *n* = 63).

**Results:**

The mean blood pressures in the two groups were well controlled throughout the study. In the high Hb group, mean Hb concentrations increased to >12 g/dL at 3 months, reaching the target range at 18 months. At the end of this study (36 months), the mean Hb concentration was 12.8 ± 0.7 g/dL in the high Hb group and 11.5 ± 1.2 g/dL in the low Hb group. The decline rate of the estimated glomerular filtration (eGFR) rate was considerably greater in the low Hb group (ΔeGFR, −5.1 ± 9.5 mL/min/1.73 m^2^) than in the high Hb group (−1.0 ± 8.4 mL/min/1.73 m^2^) (P* *=* *0.02). Of note, only a few high Hb patients developed cardiovascular events and returned to hemodialysis, but the low Hb patients did not.

**Conclusion:**

This prospective study suggests that correcting anemia to the target Hb level range (12.5–13.5 g/dL) slows renal function deterioration by >3 years in the chronic phase of allograft nephropathy.

## INTRODUCTION

The kidneys are highly susceptible to intrinsic oxidative stress resulting from systemic ischemia and subsequently to an excessive inflammatory response [1]. The pathophysiology of renal ischemia forms a vicious cycle with various clinical features, including anemia, hypertension, cardiovascular disease (CVD) and chronic kidney disease (CKD). Therefore, anemia after kidney transplantation (KTx), which is caused by certain critical factors for graft dysfunction, such as the use of immunosuppressive and antihypertensive drugs and failing erythropoietin synthesis, is considered a major determinant for the development of chronic allograft nephropathy [2, 3]. Indeed, its incidence rate has been reported to reach 45% in the first year after KTx [4]. Furthermore, anemia itself has been strongly linked to cardiovascular system damage through the complex interaction of multiple vascular effectors [5]. Correction of anemia is needed to maintain graft function, as well as cardiac function, and to improve patient survival.

Some observational studies to date have emphasized that post-transplant anemia (PTA) is closely associated with graft function and mortality [6–8]. However, there is increasing evidence that PTA does not solely reflect impaired graft function and mortality [9, 10]. Thus, the impact of PTA on graft function and mortality remains controversial. It is particularly noteworthy that KTx recipients show impaired renal function in proportion to the severity of anemia [8]. An elegant study by Choukroun and colleagues [11] showed that targeting hemoglobin (Hb) levels to >13 g/dL prevented deterioration of chronic allograft nephropathy [11]. However, the Kidney Disease: Improving Global Outcomes (KDIGO) Clinical Practice Guideline for anemia indicates that increasing Hb values from 11.5 to 13 g/dL has to be weighed against the probability of greater harm in CKD patients. Indeed, Heinze *et al*. [12] demonstrated in a retrospective cohort study that increasing Hb levels to >12.5 g/dL with erythropoiesis-stimulating agents (ESAs) in KTx recipients was associated with increased mortality, and this effect was significant at concentrations >14.0 g/dL [12].

A 3-year follow-up study of an open-label, multicenter, randomized controlled trial involving KTx recipients was conducted to test our hypothesis that sustained maintenance of target Hb concentrations at high levels (12.5–13.5 g/dL) with ESA would limit deterioration of kidney function compared with low target levels (10.5–11.5 g/dL).

## MATERIALS AND METHODS

### Study population

A total of 127 patients were enrolled from January 2012 to March 2014 at Nagoya Daini Red Cross Hospital and Masuko Memorial Hospital. The following were the inclusion criteria: 20–70 years of age; KTx performed at least 12 months earlier; stable kidney function [serum creatinine (Cr) variation <15% in patients with an estimated glomerular filtration rate (eGFR) of 15–50 mL/min/1.73 m^2^ of body surface area] for the preceding 3 months before registration; and Hb levels of 9.0–11.5 g/dL. The exclusion criteria included: urinary protein >1 g/day; history of acute rejection (AR) within the preceding 3 months; uncontrolled hypertension; infection; prior diagnosis of malignancy; iron deficiency anemia; hemorrhagic diathesis; Hb levels <10 g/dL with ESA; pregnancy or lactation; history of coronary artery disease and/or CVD; and critical allergy. Patients with Hb levels <10 g/dL on ESA therapy were eventually excluded since they may have potentially developed or currently had causative factors such as infection, chronic inflammation and hematopoietic disorders. At each visit to the hospital during the experimental period, the enrolled patients did not have the possible complications causing resistance to ESA.

All procedures were performed in accordance with the ethical standards of the institution at which the studies were conducted (IRB approval number, 20110524-2) and with the 1964 Declaration of Helsinki and its later amendments or comparable ethical standards. Informed consent was obtained from all individual participants included in the study. This study was registered with the University Hospital Medical Information Network ID: UMIN 000009594.

### Study design and intervention

**FIGURE 1:**
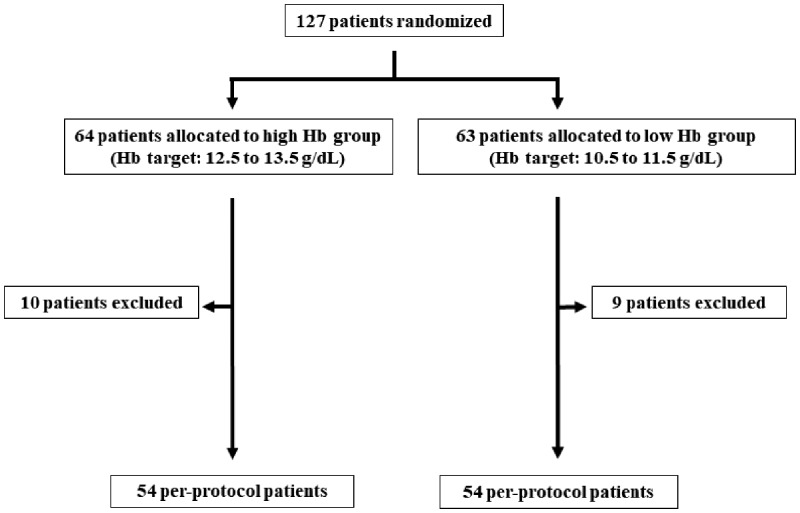
Study protocol.

As shown in Figure 1, eligible patients were randomly assigned (1:1 ratio) to achieve target Hb levels of 12.5–13.5 g/dL (high Hb group) and 10.5–11.5 g/dL (low Hb group). Randomization was stratified by age, sex, eGFR (≥30 or <30 mL/min/m^2^) and diabetes mellitus (DM) or non-DM using a computer program. Patients were followed up for 36 months. The patients visited their hospitals every 4–6 weeks and were administered an ESA (darbepoetin alfa or epoetin beta pegol) either intravenously or subcutaneously if needed. The dosage of ESA was adjusted according to the changes in Hb levels. According to the KDIGO Clinical Practice Guideline for anemia, iron supplements such as oral ferric citrate and intravenous saccharated ferric oxide were added to maintain transferrin saturation (TSAT) rates >20% or serum ferritin levels >100 ng/mL. Blood pressure (BP) was measured at the start of this study and at each visit. Antihypertensive agents were used to achieve systolic BP <130 mmHg and diastolic BP <80 mmHg according to the clinical guideline of the Japanese Society for Clinical Renal Transplantation.

### Endpoints

The primary efficacy endpoint was the difference in the rate of decline of Cr-based eGFR (eGFR_cre_) between the initiation point and the 36-month follow-up assessment. Secondary outcomes included number of patients who progressed to end-stage renal disease (ESRD), occurrence of AR and cardiovascular events during the follow-up period.

### Measurements of kidney function (eGFR_cre_, eGFR_cys_ and eGFR_ave_)

eGFR_cre_ was calculated using the equation published by the Japanese Society of Nephrology [13]: 194 × (age)^−0.287^ × (sCr)^−1.094^, including a correction factor of 0.739 for women. Cystatin C-based eGFR (eGFR_cys_) was also calculated using the equation published by the Japanese Society of Nephrology [14]: (104 × cystatin-C^−1.019^ × 0.996^age^) − 8 for men; (104 × 0.929 × cystatin-C^−1.019^ × 0.996^age^) − 8 for women. Averaged eGFR (eGFR_ave_) was calculated as the averaged value of eGFR_cre_ and eGFR_cys_. Our previous study demonstrated that eGFR_ave_ may be suitable for accurately reflecting estimated kidney function in Japanese recipients with KTx [14]. In this study, therefore, eGFR_cre_ and eGFR_ave_ were assessed.

### Statistical analysis

This study examined a continuous response variable in independent control and experimental subject groups with one control per experimental subject. In our unpublished study, the response within each subject group was normally distributed with an SD of 10. If the true difference in eGFR between the two groups was 5.7, then with a type I error (α) set at 5% in a two-sided approach and a type II error (β) set at 20% during 5-year follow-up, 50 patients in each group, for a total of 100 patients, were needed. The estimated dropout/lost to follow-up rate was 20%. An interim analysis was planned after 3 years. If there was a significant difference in the deterioration of renal function between the two groups, then the trial was to be stopped for ethical reasons. Data are expressed as means ± SD, medians with quartile values (25–75%) or numbers with percentages. The differences in continuous variables between the two groups were compared using unpaired *t*-tests or the Mann–Whitney test according to the data distribution (normal or not). Differences in categorical variables between the two groups were investigated by Fisher’s exact test. The differences in continuous variables between baseline and each follow-up point were compared by paired *t*-test or the Wilcoxon signed-rank test according to the data distribution (normal or not). All statistical analyses were performed using SPSS software, version 24 (SPSS, Chicago, IL, USA). A value of P* *<* *0.05 was considered significant.

## RESULTS

### Patient characteristics

A total of 127 patients (50.4% male) were randomly assigned to either the high Hb group (*n* = 64) or the low Hb group (*n* = 63). Table 1 summarizes the characteristics of each group. All participants were Japanese, aged 47.9 ± 12.0 (mean ± SD) years. The mean time from KTx was 112.8 ± 99.8 months, with all but one being first-time KTx recipients. Except for a significant difference in corrected calcium levels, the baseline characteristics of the enrolled patients were similar in the two groups. Most participants were taking concomitant medications in combination with immunosuppressive agents and antihypertensive drugs throughout the study period. No significant differences in the frequencies of these medications between the two groups were observed throughout the study period.

**Table 1:** Characteristics of study participants stratified by targeted Hb level

	High Hb group (*n* = 64)	Low Hb group (*n* = 63)
Male sex, *n* (%)	31 (48.4)	33 (52.4)
Age, years	47.4 ± 11.1	48.5 ± 13.0
Body mass index, kg/m^2^	21.5 ± 3.6	20.8 ± 4.1
Preemptive kidney transplantation, *n* (%)	14 (21.9)	18 (28.6)
Duration of dialysis, months	21 (10–95)	22 (12–82.5)
Duration after KTx, months	119.9 ± 96.2	106.6 ± 103.2
Cause of chronic kidney disease, *n* (%)		
Diabetic nephropathy	3 (4.5)	2 (3.2)
BP, mmHg		
Systolic	126.4 ± 10.5	126.4 ± 8.9
Diastolic	76.1 ± 8.8	76.3 ± 11.1
Laboratory examinations		
Hemoglobin, g/dL	11.4 ± 0.7	11.2 ± 0.7
Serum creatinine, mg/dL	1.59 ± 0.42	1.59 ± 0.46
Cystatin C, mg/L	1.83 ± 0.44	1.82 ± 0.49
eGFR_cre_, mL/min/1.73 m^2^	34.9 ± 8.9	36.3 ± 9.5
eGFR_cys_, mL/min/1.73 m^2^	41.4 ± 13.0	42.2 ± 13.8
eGFR_ave_, mL/min/1.73 m^2^	38.2 ± 10.0	39.3 ± 10.8
eGFR by MDRD, mL/min/1.73 m^2^	35.2 ± 9.5	36.2 ± 9.5
Fe, μg/dL	91.2 ± 29.4	87.9 ± 32.1
TIBC, μg/dL	276.1 ± 33.5	279.9 ± 42.1
Ferritin, ng/mL	92 (63–129)	84 (56–143)
TSAT, %	33.4±11.2	32.1±13.1
Bicarbonate, mmol/L	24.5±7.1	23.7±2.4
Intact parathyroid hormone, pg/mL	90.1±44.0	77.9±38.1
Corrected calcium, mg/dL	9.3±0.5	9.1±0.4
Phosphate, mg/dL	3.3±0.6	3.3±0.5
Immunosuppresive drugs		
Cyclosporine use, *n* (%)	54 (84.3)	54 (85.7)
Tacrolimus use, *n* (%)	10 (15.6)	9 (14.3)
Mycophenolate mofetil use, *n* (%)	46 (71.9)	44 (69.8)
Mizoribine use, *n* (%)	5 (7.8)	5 (7.9)
Everolimus use, *n* (%)	6 (9.3)	7 (11.1)
Antihypertensive drugs		
Angiotensin II receptor blocker use, *n* (%)	49 (76.6)	45 (71.4)
Other antihypertensive drugs, *n* (%)	25 (39.0)	27 (42.9)

The values are shown as *n* (%), mean ± SD, median (interquartile range) or numbers (%).

MDRD: Modification of Diet in Renal Disease, TIBC: total iron binding capacity.

The trial profile for the study populations is shown in Figure 1. By the end of the experiment, 10 patients withdrew from the high Hb group and 9 withdrew from the low Hb group. Withdrawals were due to chronic active antibody-mediated rejection, AR by noncompliance, general fatigue, recurrent severe diarrhea, return to hemodialysis, pulmonary embolism (PE)/deep venous thrombosis (DVT), carcinoma, blood transfusion requirement, pregnancy, calcineurin inhibitor toxicity, recurrent renal amyloidosis and change of residence. Therefore, the per-protocol population included 108 patients (54 patients in each group). As shown in Table 2, hemodialysis was required in only one patient in the high Hb group during this study, but not in the low Hb patients.

**Table 2:** Reasons for dropouts or lost to follow-ups

High Hb group (*n* = 10)		Low Hb group (*n* = 9)	
Chronic active antibody-mediated rejection	3	Chronic active antibody-mediated rejection	2
Gastric cancer	1	Blood transfusion requirement	2
AR due to noncompliance	1	AR due to noncompliance	1
General fatigue due to ESAs	1	Pregnancy	1
Recurrent severe diarrhea	1	Calcineurin inhibitor toxicity	1
Return to hemodialysis	1	Recurrent renal amyloidosis	1
PE/DVT	1	Change of residence	1
Renal cell carcinoma	1		

### Treatment of anemia

**FIGURE 2:**
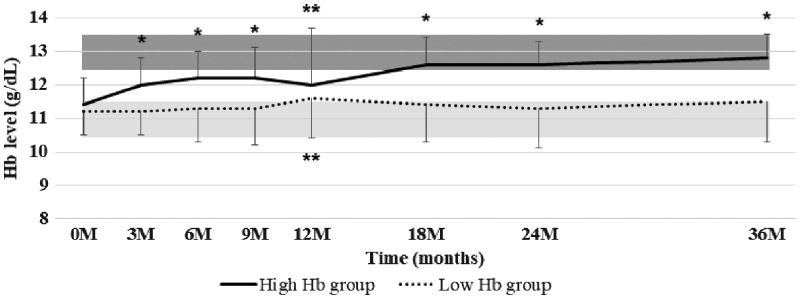
The changes in Hb levels stratified by target Hb range throughout the study period. The graph shows the means ± SD. *P < 0.001, **P < 0.05 versus the initiation time point.

**FIGURE 3:**
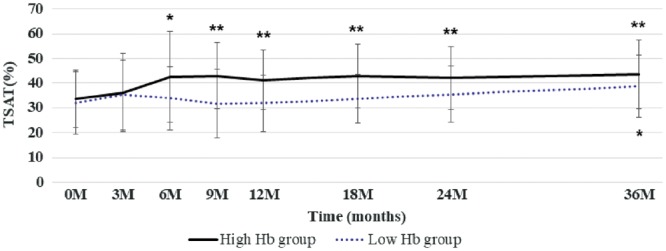
Changes in TSAT rates throughout the study period. Data are shown as means ± SD. *P < 0.01, **P < 0.001 versus the initiation time point.

The changes of the mean Hb levels in the high Hb group and low Hb group during the 36-month period are shown in Figure 2. At baseline, the mean Hb level was 11.4 ± 4.7 g/dL in the high Hb group and 11.2 ± 4.7 g/dL in the low Hb group. If needed, ESA was administered throughout the study period. In the high Hb group, the mean Hb concentration increased to >12 g/dL at 3 months, reaching the target range at 18 months. These values were then maintained until the end of the study. Meanwhile, the mean Hb concentration in the low Hb group stayed within the target range throughout the study period. At the end of this study (36 months), the mean Hb concentration was 12.8 ± 0.7 g/dL in the high Hb group and 11.5 ± 1.2 g/dL in the low Hb group. With the ratio of the titers of darbepoetin alfa and epoetin beta pegol as 1:1.2 [15], the mean ESA dosage per month was significantly higher in the high Hb group than in the low Hb group at each fiscal year (Table 3). Intravenous ESA was given to 38.9 and 25.9% of the high Hb group and low Hb group, respectively. The changes of TSAT rates in the two groups were well controlled and maintained at >30% in response to iron supplements during the 36-month follow-up period (Figure 3).

**Table 3:** Mean monthly dose of ESAs during this study

	High Hb group (*n* = 54)	Low Hb group (*n* = 54)	P*-*value
Baseline, 12 months	75.8 ± 41.4	39.2 ± 34.8	<0.001
24 months	65.3 ± 40.0	30.7 ± 34.5	<0.001
36 months	70.6 ± 38.6	39.8 ± 54.2	<0.001

Data are shown as means ± SD (µg per month).

### Renal function

**FIGURE 4:**
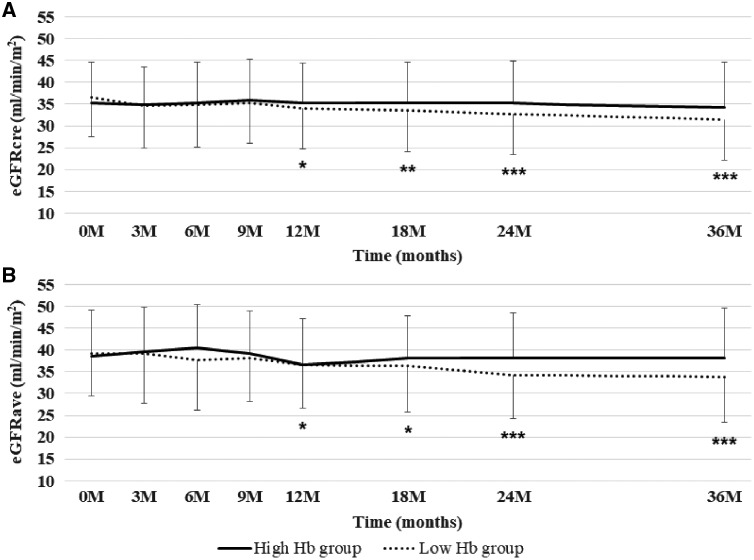
Changes in eGFR_cre_ (**A**) and eGFR_ave_ (**B**) levels. Data are shown as means ± SD. *P < 0.05, **P < 0.01, ***P < 0.001 versus the initiation time point.

**FIGURE 5:**
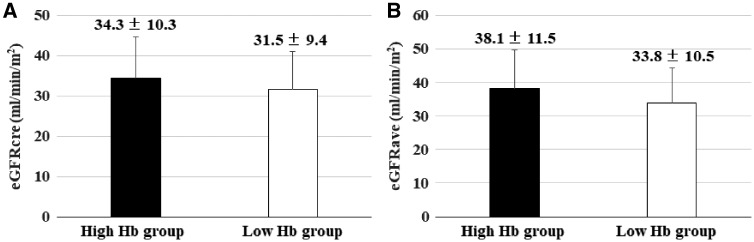
eGFR_cre_ (**A**) and eGFR_ave_ levels (**B**) stratified by target Hb range at 36 months after initiation.

**FIGURE 6:**
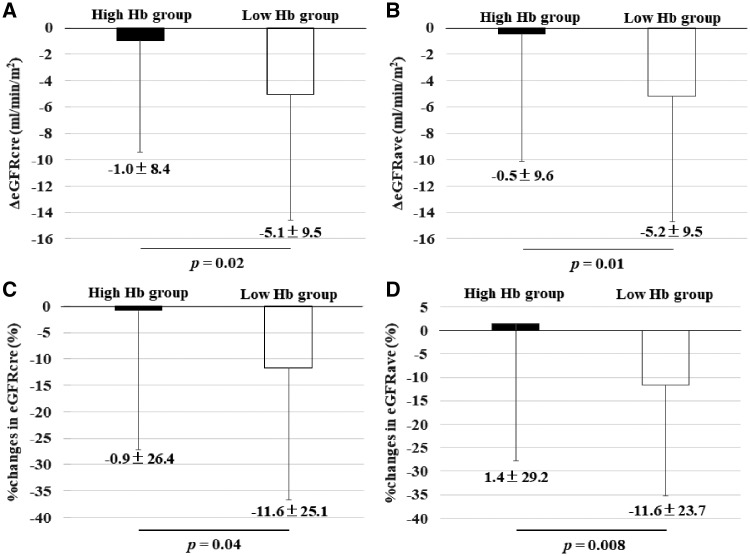
Differences between the initiation and the last time point in this study determined by ΔeGFR in eGFR_cre_ (**A**) and eGFR_ave_ (**B**), and %changes in eGFR_cre_ (**C**) and eGFR_ave_ (**D**) GFR.

At baseline, the mean eGFR_cre_ was 34.9 ± 8.9 mL/min/1.73 m^2^ in the high Hb group and 36.3 ± 9.5 mL/min/1.73 m^2^ in the low Hb group, and the mean eGFR_ave_ was 38.2 ± 10.0 mL/min/1.73 m^2^ and 39.3 ± 10.8 mL/min/1.73 m^2^, respectively. While eGFR_cre_ and eGFR_ave_ in the high Hb group were maintained throughout the study period, both parameters in the low Hb group showed gradual decreases with significant differences after 12 months (Figure 4). At the end of anemia correction, the mean eGFR_cre_ was 34.3 ± 10.3 mL/min/1.73 m^2^ in the high Hb group and 31.5 ± 9.4 mL/min/1.73 m^2^ in the low Hb group, and the mean eGFR_ave_ was 38.1 ± 11.5 mL/min/1.73 m^2^ and 33.8 ± 10.5 mL/min/1.73 m^2^, respectively (Figure 5). The difference in eGFR_cre_ between baseline and 36 months (ΔeGFR_cre_) was −1.0 ± 8.4 mL/min/1.73 m^2^ in the high Hb group and −5.1 ± 9.5 mL/min/1.73 m^2^ in the low Hb group (P* *=* *0.02), and ΔeGFR_ave_ was −0.5 ± 9.6 mL/min/1.73 m^2^ and −5.2 ± 9.5 mL/min/1.73 m^2^ (P* *=* *0.01), respectively. Likewise, %changes in eGFR_cre_ were −0.9 ± 26.4% in the high Hb group and −11.6 ± 25.1% in the low Hb group (P* *=* *0.03), and those of eGFR_ave_ were 1.4 ± 29.2 and −11.6 ± 23.7%, respectively (P* *=* *0.008) (Figure 6). There were no significant differences in ΔGFR_cre_ by the route of administration of ESA in the respective groups (data not shown).

### ESRD, AR and vascular diseases

At the end of this study, only one patient (1.6%) in the high Hb group was returned to hemodialysis due to ESRD (Table 2). AR occurred in one patient (1.6%) in each group, and chronic active antibody-mediated rejection was observed in three patients (4.7%) in the high Hb group and two patients (3.2%) in the low Hb group. According to the criteria established at the Banff 2013 meeting [16], the diagnosis of rejection was clinically and pathologically performed using light microscopy and immunofluorescence by expert nephropathologists. PE/DVT occurred in one patient on oral contraceptive pills in the high Hb group. No coronary and peripheral vascular diseases were found during this study.

### Control of BP

**FIGURE 7:**
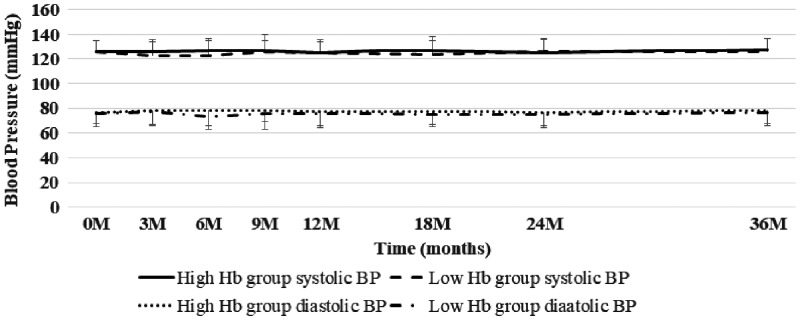
Changes in BP in the high Hb group and the low Hb group.

At baseline, the mean systolic BP was 126.4 mmHg in both groups, and the mean diastolic BP was 76.1 mmHg in the high Hb group and 76.3 mmHg in the low Hb group (Figure 7). The antihypertensive drugs showed a similar profile between the groups. The mean, systolic BP and diastolic BP in the two groups were well controlled during the 36-month follow-up period. In two patients in the high Hb group, BP increased transiently to >160/90 mmHg in response to a step-by-step rise of the Hb level, but BP returned to within the normal range with control Hb levels.

### Safety

Adverse events occurred in 50.0 and 57.1% in the high Hb group and low Hb group, respectively (Table 4). A total of 26 patients (20.5%) were hospitalized: 11 (17.2%) in the high Hb group and 15 (23.8%) in the low Hb group. Only one patient (1.6%) in the high Hb group had a serious adverse event, PE/DVT, but eventually made a complete recovery.

**Table 4:** Frequency of adverse events at the end of this study

Adverse event	High Hb group (*n* = 64)	Low Hb group (*n* = 63)
Patients requiring of hospitalization, *n* (%)	11 (17.2)	15 (23.8)
Patients with adverse events, *n* (%)	32 (50.0)	36 (57.1)
Chronic active antibody-mediated rejection, *n* (%)	3 (4.7)	2 (3.2)
AR due to noncompliance	1 (1.6)	1 (1.6)
Cardiovascular disorders, *n* (%)		
PE/DVT	1 (1.6)	0
*De novo* hypertension	3 (4.7)	0
Infections, *n* (%)		
Upper respiratory tract infection	8 (12.5)	10 (15.9)
Pneumonia	1 (1.6)	2 (3.2)
Urinary tract infection	2 (3.1)	1 (1.6)
Bacteremia	1 (1.6)	2 (3.2)
Gastrointestinal disorders, *n* (%)	6 (9.4)	7 (11.1)
Symptoms, *n* (%)		
Palpitation	2 (3.1)	0
Chest discomfort	1 (1.6)	0
Headache	2 (3.1)	0
Dizziness	1 (1.6)	0

## DISCUSSION

The present study results suggest that correction of anemia, along with conventional immunosuppressive and antihypertensive medications as needed, slows deterioration of kidney function by >3 years in the chronic phase of allograft nephropathy. The setting of the target Hb concentration ranges was intended to make it difficult to develop CVD. Akizawa and colleagues have investigated the beneficial effects of maintaining high Hb levels (11.0–13.0 g/dL) on kidney function and the risk of CVD in predialysis patients with CKD [17, 18]. In the present study, anemia was treated with darbepoetin alfa or epoetin beta pegol, which have longer half-lives. The rate of decline of eGFR_cre_, which was evaluated as the primary efficacy endpoint, was considerably greater in the low Hb group (%changes, −11.6% ± 25.1%/3 years) than in the high Hb group (−0.9% ± 26.4%/3 years). Besides maintenance of kidney function by correction of anemia to the high Hb range throughout the study duration, of note, few adverse events related to CVD occurred in the population of this group.

Several observational and retrospective studies have provided conflicting conclusions on whether correction of anemia in KTx recipients would be beneficial in preventing the development of chronic allograft nephropathy or be deleterious in the production of cardiovascular events [4, 6–11, 19]. Although the subjects were patients with CKD before dialysis, the large-scale comparative clinical trials, such as the CHOIR, CREATE, TREAT and ACORD studies, demonstrated negative effects on kidney function and cardiovascular events by correcting anemia [20–23]. In the CHOIR study, especially the incidences of ESRD requiring starting dialysis and cardiovascular events were higher in the high Hb group (mean Hb, 13.5 g/dL). The discrepancy may be explained by the speculation that chronic allograft kidneys have superiority from the viewpoints of histopathology, hemodynamics and immune biology when compared with the kidneys in predialysis CKD patients. However, the effectiveness of correcting anemia by ESA would extend not only to graft kidneys, but also to native kidneys [24–26]. Taking into consideration the undesirable results of the aforementioned studies regarding the efficacy and safety of anemia correction, we attempted to gradually increase the Hb values to the target Hb concentration range by adjusting the ESA dosage. Although the mean Hb levels reached the target range at 18 months after the initiation of this study, our goal was successfully achieved in the remaining 18 months. This may be one of the reasons why serious adverse events such as CVD did not occur in the present study. Similar to the present data, a prospective study by Choukroun *et al*. [11] also demonstrated that correction of anemia by taking epoetin-β reduced the progression of chronic allograft nephropathy. In contrast to previous studies, there were notably no cardiac events and few vascular disorders in the higher Hb group (13–15 g/dL). In the present study, only one serious adverse event, PE/DVT, occurred in a patient taking oral contraceptive pills in the high Hb group, which finally resulted in complete recovery. Consequently, ESA dosages used for the high Hb group in this study were similar to those of the higher Hb group in Choukroun’s study [11]. Such contradictory conclusions regarding the onset of CVD by anemia correction may be closely associated with the inherent methodology, including target Hb levels, their speed of correction and the history of the populations.

The kidneys, rich in endothelium, are highly susceptible to intrinsic and incessant oxidative stress caused by the ischemia-reperfusion occurring during grafting and uncontrollable hypertension [27–29]. Limited oxygen delivery to the tubulointerstitium caused by anemia leads to the formation of reactive oxygen species. This further induces the release of proinflammatory molecules and profibrogenic stimulants for tubular epithelial cells and interstitial fibroblasts, subsequently leading to the development of CKD with an excessive inflammatory response. In transplant recipients particularly, the hypoxic damage may be more potentiated by the use of long-term immunosuppressive agents such as calcineurin inhibitors and by the concomitant presence of congestive heart failure. There is indeed a growing body of evidence for the participation of oxidative stress in the exacerbation of allograft dysfunction and for the beneficial effects of correcting anemia on these processes [11, 30, 31]. In a study by Remuzzi *et al*., ESA itself prevented chronic allograft nephropathy through regulation of intragraft expression of antiapoptotic, antioxidant and angiogenic properties [32]. ESA treatment also improved kidney transplant outcomes through regulation of immune tolerance involving functional regulatory T cells [33]. From the perspective of these clinical and basic studies, the striking slowing of graft dysfunction would contribute to anemia correction with ESA and the direct tissue-protective effects of ESA [34–36]. As a matter of course, a higher dosage and/or resistance to ESA therapy predicts increased mortality and cardiovascular events in KTx recipients [37]. We emphasize that significant differences in graft function between the two groups were observed at 18 months after study initiation with Hb ≥12.5 mg/dL.

This study’s results must be interpreted with several cautionary notes regarding the study methodology and the size of the study population. This population consisted of 127 Japanese participants only and showed lower body mass index values compared with Europeans and Americans resulting from differences in lifestyle and physique. These differences might affect this study’s results. Attention is necessary to apply our findings to participants with a history of CVD, which was not suitable for the criteria of this study. Since the frequency of medications such as immunosuppressive reagents and antihypertensive drugs was not randomly divided in our population, it is unknown how this issue affects our results.

In conclusion, correction of anemia (Hb levels 12.5–13.5 mg/dL) with an ESA showing a longer half-life prevents the progression of chronic allograft nephropathy in KTx recipients, as long as BP is appropriately controlled. Furthermore, this study shows the validity and safety of correcting anemia for observational and retrospective studies in the near future. Further large-scale comparative studies would afford key insights for understanding the underlying mechanism of cardiorenal syndrome in KTx recipients, as well as supporting the present conclusions.
